# Diversity of chemical composition and nutritional value in grain from selected winter wheat cultivars grown in south-western Poland

**DOI:** 10.1038/s41598-024-53094-0

**Published:** 2024-02-01

**Authors:** Anna Szuba-Trznadel, Bernard Gałka, Joanna Kamińska, Anna Jama-Rodzeńska, Zygmunt Król, Daniel Jarki, Bogusław Fuchs

**Affiliations:** 1 Department of Animal Nutrition and Feed Science, Faculty of Biology and Animal Science, Wroclaw University of Environmental and Life Sciences, 51-630 Wroclaw, Poland; 2 Department of Applied Mathematics, Faculty of Environmental Engineering and Geodesy, Wrocław University of Environmental and Life Sciences, 50-363 Wroclaw, Poland; 3 Institute of Soil Science and Environmental Protection, Faculty of Life Sciences and Technology, Wrocław University of Environmental and Life Sciences, 50-363 Wroclaw, Poland; 4 Institute of Agroecology and Plant Production, Faculty of Life Sciences and Technology, Wrocław University of Environmental and Life Sciences, 50-363 Wroclaw, Poland; 5 Saatbau Poland Sp. z o.o., Żytnia 1, 55-300 Środa Śląska, Poland

**Keywords:** Plant sciences, Biogeochemistry, Environmental sciences

## Abstract

Given the low protein coverage by legumes in Poland, alternatives (with high protein content and high nutritional value) are being sought (with high protein content and high nutritional value of protein) that could replace these plants. Cereal cultivation dominates in Poland; hence, the search for high-value plants will also consider this group of plants. The aim of the study was to compare the nutritional value of proteins from two wheat cultivars. A field experiment conducted in Zawidowice in south-western Poland in 2019 investigated the nutritional values of two winter wheat cultivars: Aurelius and Activus. These two cultivars were compared in terms of their chemical composition, the biological value of their proteins for animal nutrition, and the content of macro- and microelements. Significant differences in chemical composition were found between the tested wheat cultivars. In terms of the chemical composition, i.e. the content of protein, fiber and ash, the Activus cultivar was characterized by significantly better parameters. This cultivar also had significantly higher gross energy. In turn, a significantly higher content of essential amino acids, i.e. lysine, cysteine, tryptophan, histidine, leucine, ioleucine, and valine, was found in the Aurelius cultivar; therefore, the indicators determining the biological value of the protein are more favorable in the Aurelius cultivars. Meanwhile, in terms of selected macro- and microelements the Auerlius cultivar was more valuable. Varietal progress is necessary to obtain cultivars with the essential nutrients needed by animals to satisfy their dietary requirements.

## Introduction

One of the main objectives of modern agriculture is to obtain a satisfactory yield of wheat grain with high values for indicators of its quality, with protein being the most important trait. Grain yield and protein content in grain are two very important characteristics of wheat. These are controlled by genetic properties, but developmental conditions also support their expression. Many varieties of winter wheat with higher protein content (2–3% higher) are not always characterized by a high nutritional value, which may be due to soil, weather, or agrotechnical conditions^1^.

Wheat (*Triticum aestivum* L.) is the most important cereal cultivated in the European Union^2^, and its grain can be used in human and animal nutrition^3–5^. Any surplus production, lower quality or small size grains, or by-products of its processing, such as wheat bran, flour, and dried wheat decoctions, are used as fodder^3^. Cereal grains (including wheat) are characterized with a low protein content compared to legumes and oilseeds. Despite the low protein content, wheat provides an appropriate amount of energy (mainly due to the high starch content), which can be used in the nutrition of monogastric animals (poultry and pigs). In addition, due to its high level of inclusion in the diet, it provides significant amounts of nutrients and minerals for animals^6^.

Analysis of the chemical composition of wheat demonstrates significant differences in the content of nutrients, minerals and anti-nutritional compounds, mainly due to the diversity of genetic and environmental factors^7,8^. Varieties of common winter wheat differ significantly in terms of grain yield and protein content. Variation in protein content that may be due to genetic differences is generally lower than variation caused by environmental influences. The protein content of wheat grains ranges between 8 and 20%^9–13^. This variability is mainly due to environmental factors (temperature, light intensity and water availability). In addition, agricultural practices, including the use of nitrogen fertilizers, can modify the level of protein in grain.

Lysine is the most deficient essential amino acid in wheat protein^9^. The effect of nitrogen fertilization on the protein content in wheat grain and the amino acid profile is dependent on the wheat cultivars used^14–16^. A decrease in the relative content of lysine in grain may result from an increase in gluten proteins, which in turn can depend on the use of excessive doses of nitrogen fertilizers^9,17^. Therefore, research in the field of genetics and genetic engineering focuses not only on increasing the level of total protein, but above all on the level of essential amino acids (while maintaining yields relative to standard cultivars)^4,5,18^. One of the aims of modern wheat breeding in Poland is to obtain cultivars of high yield, quality and nutritional value as well as resistance to diseases.

The basic protein element on the national feed market is still post-extraction soybean meal, which cannot be replaced by any other plant. However, due to the continuous deficit of feed protein and its dependence on imports, the introduction of protein raw materials with similar parameters is necessary. A better knowledge and understanding of the chemical properties, protein quality and amino acid composition of different cultivars will be beneficial not only for breeders and farmers, but also for feed producers. New genome and comparative studies with model species have recently been revealed. Moreover, alongside the additional marker selection programs (MAS), additional tools, including maps and molecular markers, have accelerated increases in protein content^19^. The use of commercial breeding for improving protein content is influenced by environmental conditions and affects the yield component, specifically with alleles derived from wild emmer^20^. The experiment presented below included two cultivars of winter wheat cultivated in a field experiment and tested for their nutritional value, including protein. The working hypothesis assumed that the protein content of the tested winter wheat cultivars would be varied and dependent on genetic properties. The aim of the study was to determine the chemical composition and amino acid profile of two winter wheat cultivars (harvested in 2020–2021).

## Materials and methods

### Agrotechnical conditions of the field experiment

A field experiment with winter wheat was conducted in Zawidowice (Bierutów commune, Oleśnica poviat) in 2019. Four fields were established, each with an area of approx. 20 ha. Two fields were sown with the Activus cultivar and the other two with Aurelius. The research objective was an assessment of the impact of standard cereal (wheat) agricultural treatments on the yield and chemical composition of these cultivars of winter wheat grain. The forecrop was winter rape.

Nitrogen fertilizer was applied three times during the growing season. The three-time application of nitrogen was applied in specific development phases, i.e., 170 kg N ha^−1^ (3/03/2020) during the start of vegetation in the spring (BBCH 22), 140 kg N ha^−1^ in the stem elongation phase (BBCH 31), and 100 kg N ha^−1^ in the heading phase (BBCH 57). Nitrogen fertilizers were applied using a Bogballe fertilizer spreader with a working width of 28 m. Phosphorus and potassium fertilization were applied according to the standard fertilization methodology for wheat, i.e. 90 kg P_2_0_5_ per ha and 100 kg K_2_O per ha. Micronutrient (foliar) fertilization was applied with OSD micro grain at a dose of 1 kg ha^−1^ (26/04/2020), OSD micro grain 1 kg ha^−1^ + urea 10 kg ha^−1^ (19/05/2020), and in the third term (05/06/2020) OSD micro grain 1 kg ha^−1^ + urea 10 kg ha^−1^. At the time of flowering (01/05/2020), Regullo 500 EC Innvigo growth regulator was used: 0.2 l ha^−1^ + ccc 720Sl 1 l ha^−1^.

Before sowing, the rapeseed stubble was disked with a Unia Grudziądz disc harrow with a working width of 6 m, followed by cultivation with a Horsch Tiger mt4 cultivator.

Wheat was sown at the optimal date for this region (October 10–12, 2019) with a PÖTTINGER TERRASEM 4c seed drill, using certified seed with known germination capacity (Activus: 95%; Aurelius: 93%) and WTS (weight of thousand seeds) (Activus: 45 g; Aurelius: 46 g). The sowing rate used was 370 pieces per m^2^.

Seeds were pretreated and sown at a depth of 3 cm, with a row spacing of 12.5 cm. Chemical protection against weeds, diseases and pests was applied in accordance with integrated pest management recommendations. Dicotyledonous and monocotyledonous weeds were controlled with Bizon (dose 1 l ha^−1^) (November 12, 2020) and Corida 20 g ha^−1^ (April 2, 2020)—a systemic foliar herbicide in the form of water-soluble granules. Fungicides were also applied on three dates: treatment T1 Promax 450EC 0.5 l ha^−1^ + Tern Premium 575EC 0.8 l ha^−1^ + micro treatment (April 26, 2020); treatment T2 durato pro 497 SC 05 l ha^−1^ + Tanzer 250 SC (May 19, 2022); and treatment T3 Syrius 250 EW 0.6 l ha^−1^ + treatment for sparviero aphids at 0.075 l ha^−1^ (June 3, 2020). The treatments were performed with a Lemken Primus sprayer with a working width of 28 m.

Harvest was performed in the full maturity phase—July 27, 2020—with a New Holland CX5.80 combine harvester. After harvest, grain yield was assessed at 14% moisture content (average yield 7.8 t ha^−1^).

### Description of cultivars used in the experiment

The research material comprised wheat grain (*Triticum aestivum* L.) from the harvest in 2020–2021, sourced from Saatbau Polska Sp. z o.o.^21^. The two cultivars came from different technological quality groups.

Winter wheat of the Activus cultivar was selected for the evaluation. The breeder for this cultivar was SZ Donau Austria, and this is a so-called class A wheat. The advantages of this cultivar are characterized by its high tolerance to water deficiency, early ripening and high protein content. The optimal sowing density is 340 grains per m^2^, and it is recommended for cultivation on soil exposed to drought. It is indicated as being resistant to many diseases as well as having a high grain yield.

The second cultivar used in the study was Aurelius, with a very high tolerance to water shortage. It is bony wheat cultivar, i.e., it possesses active photosynthetic organs that provide a significant amount of assimilates to the kernels. Their beneficial effect on yield is especially noticeable in dry years and in semi-dwarf varieties. Their positive role in inhibiting the development of aphids has also been noted. It has a well-developed root system, significant resistance to lodging, and a high protein content in the grain. The recommended sowing rate is 300–400 seeds per m^2^.

### Soil conditions during the experiment

Wheat was sown on typical lessive soil composed of light clay belonging to classes 3b (medium-good arable soils) and 4a (medium-good arable soils). The soil was characterized by a neutral soil reaction (6.6), previously regulated by liming.

Selected chemical parameters were determined by the Egner-Riehm method^22^ at the Chemical and Agricultural Station in Wrocław: P, K and Mg, pH. Soil reaction was measured with a glass pH electrode (1:5 soil:deionized water, measurement after 30 min).

The soil was characterized by an average abundance of phosphorus (13.88 mg per 100 g of soil) and potassium (18.6 mg per 100 g of soil) and a low abundance of magnesium (4.47 mg per 100 g of soil).

### Weather conditions

**Figure 1:**
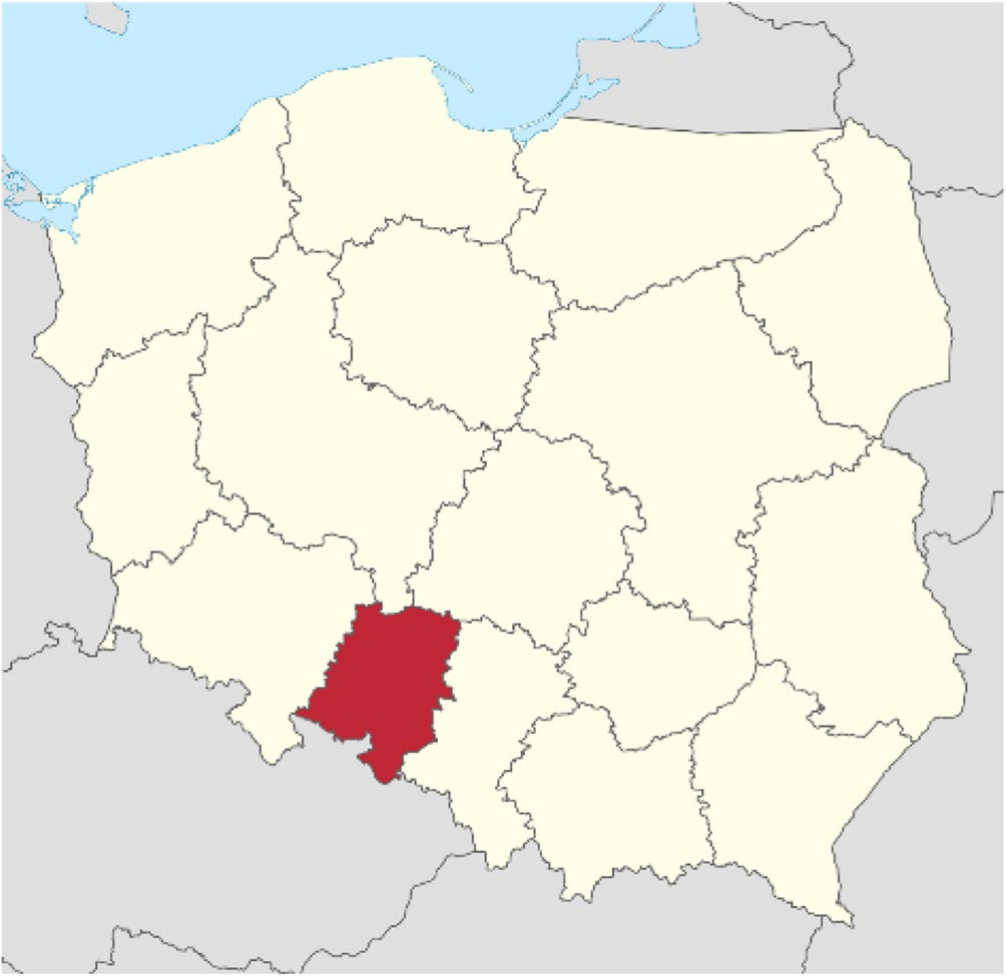
Location and aerial view of experiment fields (Zawidowice 51° 07′ 59″ N 17° 28′ 34″ E, Poland).

The experiment with winter wheat was established in Zawidowice, located in the south-western part of Poland, as presented in Fig. 1.

Based on long-term observations conducted by the Meteorological Station in Oleśnica, the average temperature for the Bierutów area is January—1.5 °C, and July—18.2 °C. Annual precipitation is recorded at the level of 610 mm. The period of occurrence of more intense rainfall is recorded from May to September. June and July are the months with the highest rainfall. The least rainy month is February. The growing season lasts on average from April 20 (April 2–May 7) to October 20 (October 4–November 7).

**Figure 2:**
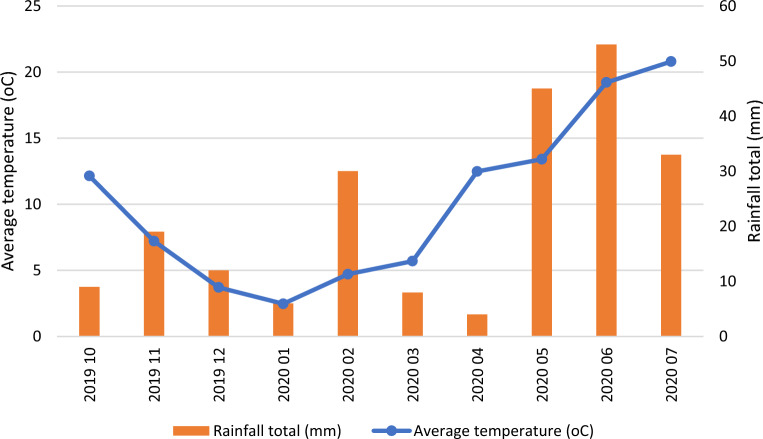
The course of weather conditions in the years of experiment 2019–2020.

The 2019/2020 season was characterized by higher temperatures compared to the average values for this region. The month with the highest temperatures was July, while the month with the lowest temperatures was January. The rainiest month was June (with 53 mm of precipitation). The lowest amount of precipitation was recorded in April (Fig. 2).

### Plant sampling and chemical analysis

Winter wheat grains after harvesting were sampled for chemical analysis. Before analysis, the grain was dried and ground.

The basic chemical composition of wheat grain (dry matter, crude ash, total protein, crude fiber) was determined using the AOAC^23^ standard method:

- Dry matter (DM, AOAC: 934.01) by the gravimetric method at 105 °C for 4 h in accordance with the Polish standard;
- Crude protein (CP, Kjeldahl method, AOAC: 984.13)—crude protein content was analyzed by multiplying the percentage of nitrogen (N%) determined in the sample. Nitrogen was determined on a Kjeltec 2300 Foss Tecator (AOAC: 984.13) by multiplying the result by the factor (6.25). In order to reliably verify the coverage of an animal's demand for protein, it is necessary to determine the "true protein" (TP), defined as protein nitrogen (PN) obtained after separating the non-protein nitrogen (NPN) fraction from the total nitrogen (TN);
- Crude ash (CA, AOAC: 942.05)—crude ash formed as a result of burning the sample in a muffle furnace (Czylok, Jastrzębie-Zdrój) at a temperature of 550 °C for 24 h;
- Ether extract (EE, Soxhlet method involving diethyl ether extraction, AOAC: 920.39A) using a BUCHI Extraction System B-811 (BÜCHI, Flawil, Switzerland);
- Crude fiber (CF) by the Hennenberg-Stohman method (AOAC: 978.10) using a Fibertec Tecator Foss laboratory analysis apparatus. The mineralization of the samples was determined with a Mars 5 microwave digestion system version 194A06 (CEM Corporation, Matthews, NC, USA) using HNO_3_. Neutral Detergent Fiber (NDF) and Acid Detergent Fiber (ADF) levels were determined according to the methods of Van Soest (1991) using an Ankom 200 fiber analyzer (Ankom Technology Corporation, NY, USA);
- The gross energy (GE) of the grains was determined in a KL-11 "Mikado" bomb calorimeter (Precyzja-Bit Sp. z o.o., Bydgoszcz, Poland);
- The content of nitrogen-free compounds (NFE) was calculated based on the difference according to the formula:
  $${\text{NFE}} = {\text{dry}}\;\;{\text{matter }} - \left( {{\text{total}}\;\;{\text{protein}} + {\text{crude}}\;\;{\text{fiber}} + {\text{crude}}\;\;{\text{fat}} + {\text{crude}}\;\;{\text{ash}}} \right)$$
- Amino acids (AA) were determined by ion exchange chromatography using an AAA 400 amino acid analyzer (INGOS, Prague, Czech Republic) according to AOAC standard protocol (AOAC: 994.12). Tryptophan was determined using a 2000 RS spectrophotometer (Aqualytic, Dortmund, Germany) at 590.0 nm (AOAC: 988.15);
- Macro- and microelement content:
  A. Nitrogen by the Kjeldahl method, P by the vanadomolybdate method, magnesium with titanium yellow, and potassium and calcium on a flame photometer (BWB Technologies UK Ltd., Newbury, UK) using flame photometry. The plant material was mineralized with sulfuric acid and perhydrol in an electric furnace at 400 °C;
  B. Micronutrients by flame atomic absorption spectrophotometry on a Spectra AA 200 apparatus by Varian. The required conditions regarding wavelength, gap width and flame height were used to determine each of the tested micronutrients;
- Evaluation of the nutritional value of protein: the chemical score of restriction amino acids (CS), the essential amino acid index (EAAI), and the protein efficiency ratio (PER) were used to assess the nutritional value of the protein.

The chemical result of the restriction amino acid—CS—was calculated on the basis of the formula given by Block and Mitchell^24^, which consists in determining the ratio of the content of the essential limiting amino acids of the test protein (ab) to the content of the same amino acid in the reference protein (aa).

$$CS = ab\div aa \times 100$$

In our research, we used whole egg protein standards (WE) as amino acid standards.

The essential amino acid index (EAAI) according to Oser^25^ was calculated as the geometric mean of all EAAs and histidines related to the content of these amino acids in the reference protein (in g per 100 g CP). The index was calculated based on the formula:

$$EAAI = 10{\text{log}}(EAAI)$$

The predicted protein efficiency ratio (PER) was calculated using the regression equations given by Alsmeyer et al.^26^

$$PER1 = -0.684 + 0.456 \times Leu- 0.047 \times Pro$$

$$PER2 = - 0.468 + 0.454 \times Leu- 0.105 \times Tyr$$

$$PER3 = - \mathrm{1,816} + 0.435 \times Met + 0.780 \times Leu + 0.211 \times His - 0.944 \times Tyr$$

The biological value of the protein (BV) was calculated according to the equation given by Oser^27^

$$BV = 1.09 (EAA) - 11.7$$

### Statistical analysis

The results of the experiment were subjected to statistical analysis. Basic descriptive statistics were determined. Due to the small size of the data set and the inability to verify the hypothesis of the compatibility of the distribution of features with the normal distribution, the comparison of cultivars was performed using the non-parametric Mann–Whitney U test at the significance level α = 0.05 (Mann and Whitney)^28^. The calculations were conducted using Statistica 13^29^, with graphics also using the R package (Cran R).

## Results

### Nutritional value of selected wheat cultivars

**Figure 3:**
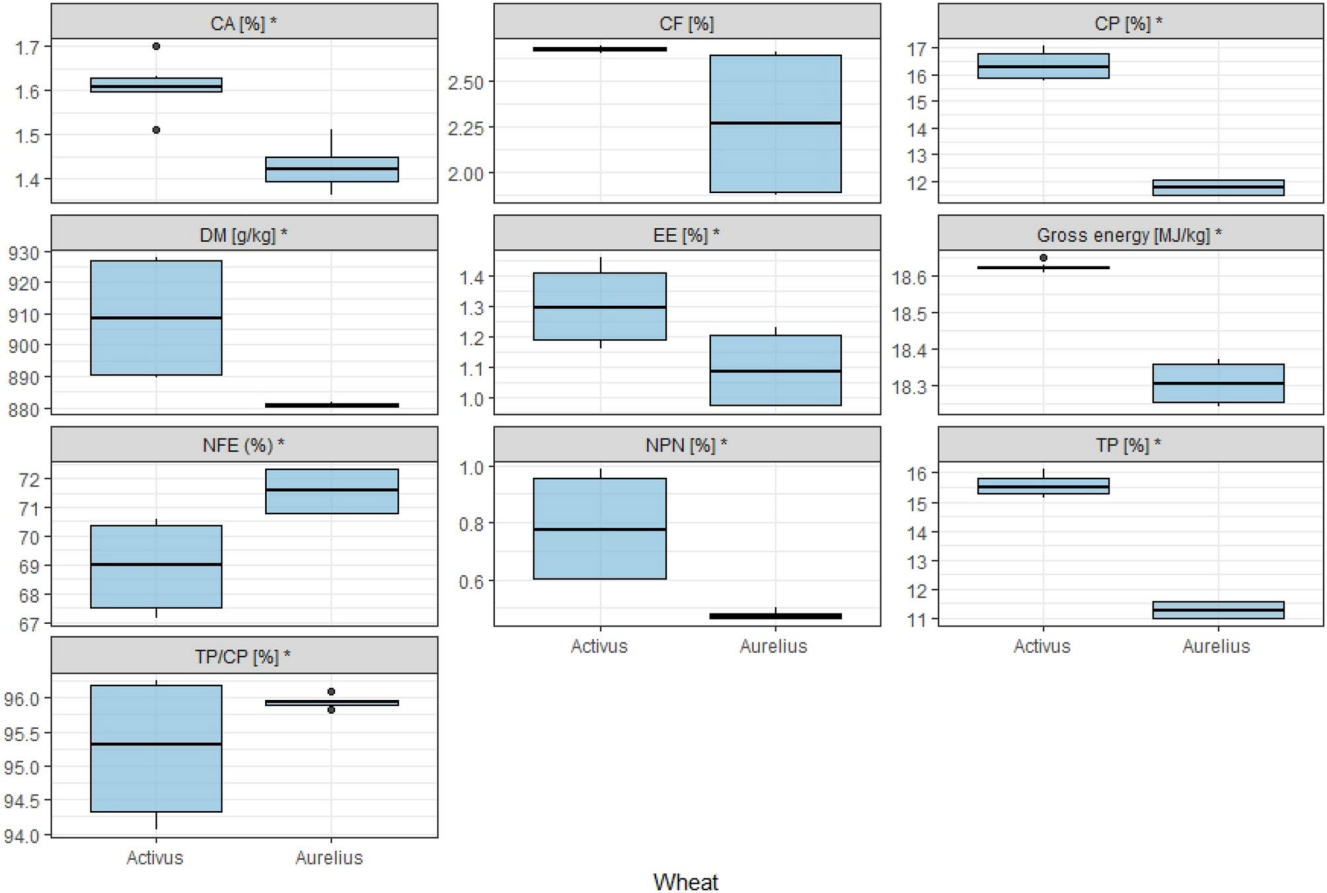
Chemical composition of wheat grains. *DM* dry matter, *CA* crude ash, *CP* crude protein, *TP* true protein, *NPN* non-protein nitrogen fraction, *CF* crude fibre, *EE* ether extract, *NFE* nitrogen-free extracts, *GE* gross energy, *statistically significant difference between cultivars of wheat.

One of the essential nutrients of cereals is protein, which is the basic criterion for assessing the raw material and its usefulness in feeding monogastric animals. The level of CP (Table 1S, Fig. 3) in wheat grain of the studied cultivars varied: from 117.5 to 163.4 g kg^−1^. The highest significant content was obtained in cv. Activus (163.4 g kg^−1^). The content of CP in Activus wheat was significantly (4.6%) higher compared to Aurelius (*p* < 0.01). The share of TP in CP amounted to 96% in Aurelius grain, while in Activus grain this value was 95%. Thus, the TP level was 16% in Activus. This value was 38% higher than the amount in the Aurelius cultivar. This high content of CP (including TP) in Activus wheat creates great opportunities for its use in compound feed mixtures for monogastric animals.

Fat content mainly determines the energy contained in wheat. The Activus cultivar was characterized by significantly higher energy values (GE) in the tested grains. Aurelius wheat was observed to have circa 2% lower energy concentration than Activus (Fig. 3). The conducted tests showed that the content of CF in the tested cultivars varied. Aurelius contained significantly less CF than did Activus (Fig. 3). Crude ash (CA) content (indicating the amount of minerals) also significantly differentiated the cultivars, averaging 1.6% and 1.4% for Activus and Aurelius, respectively (Fig. 3).

### Amino acid composition of protein

**Figure 4:**
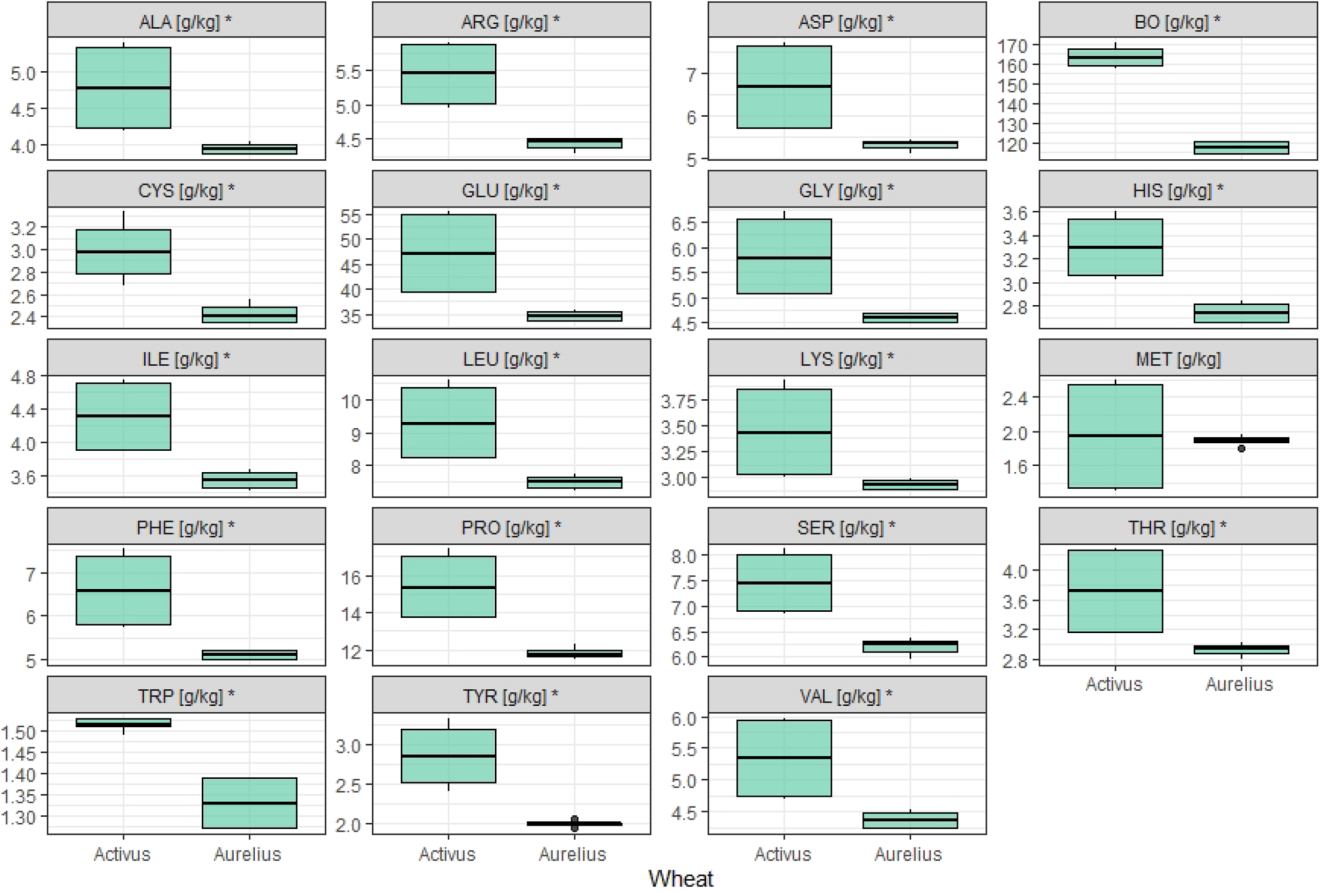
Amino acid composition of selected wheat grains. *statistically significant difference between cultivars of wheat.

When balancing feed mixtures for monogastric animals, it is necessary to both consider essential amino acids (EAA, including LYS, MET, THR, TRP, VAL and ILE) and examine the protein nutritional value. The content of amino acids (AA) in the cereal raw material results from the amount of protein. The sum of all amino acids (Ʃ AA) and the sum of essential amino acids (Ʃ EAA) were significantly higher in the grain of cv. Activus compared to cv. Aurelius (*p* < 0.01). The essential amino acids constituted ca. 33% (in cv. Activus) and 34% (in cv. Aurelius) of the total AA. In the tested samples of cv. Activus, the AA content was higher (TRP 12%, LYS 15%, THR, VAL, ILE, LEU, HIS, CYS ca. 20%, and TYR even 30%) than in cv. Aurelius (Table 1S, Fig. 4). The results indicate that this raw material could supplement AA deficiencies in feed mixtures, depending on its amount in the diet.

The dominant AAs in wheat are PRO and GLU. The sum of these amino acids is 63 g kg^−1^ (in cv. Activus) and 47 g kg^−1^ (in cv. Aurelius), constituting 45% and 43% of the AA, respectively. Per 100 g of CP, the total content of PRO and GLU was 38 g and 40 g, respectively. There were statistical differences in the shares of PRO and GLU in CP (%) with higher shares in Aurelius cultivar. In Aurelius, the share of PRO in CP was 10% and GLU in CP 30%. These values were 7% and 2% higher, respectively, than the amounts in Activus (Table 1S, Fig. 4).

Each AA showed similar characteristics for the considered measurement units for cv. Aurelius. Slightly different were TYR, characterized by very low variability (the coefficient of variability (cv) amounted to 1.8%) and MET (cv = 4.9%). A much greater differentiation between the characteristics for the discussed measurements was shown by wheat of cv. Activus. The most prominent among the other amino acids were TRP (with the smallest cv = 1%), MET (with the highest cv = 34.4%) and CYS (cv = 8.8%) (Fig. 4).

**Figure 5:**
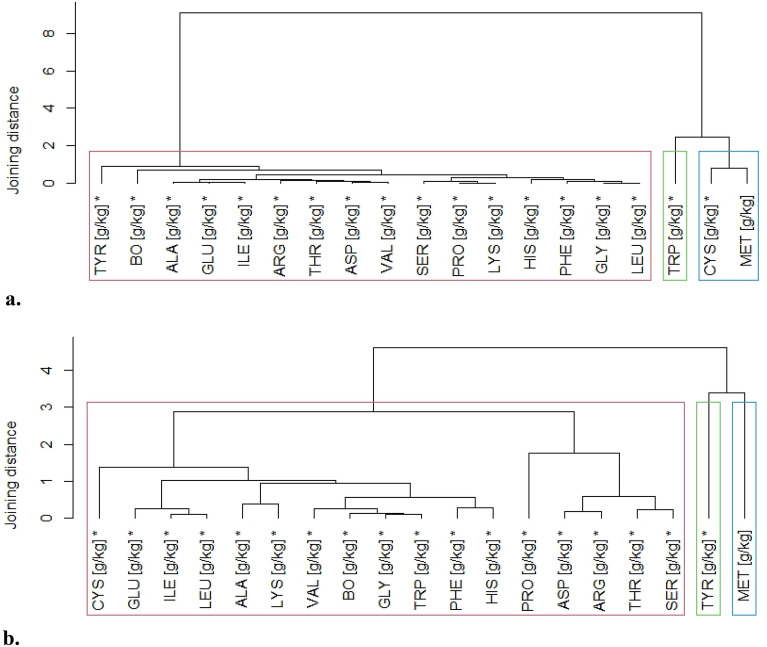
(**a**) Amino acid composition of Activus wheat grains, (**b**) Amino acid composition of Aurelius wheat grains, *statistically significant difference between amino acids.

The collected data are also presented using hierarchical tree diagrams (Figs. 5a,b). Notably, there is a definitely greater differentiation between the amount of TRP, CYS and MET and the remaining amino acids in cv. Activus, which was reflected in the hierarchical tree diagrams in the form of separate branches (Fig. 5a). In contrast, CYS did not stand out among the listed amino acids in cv. Aurelius (Fig. 5b).

### Nutritional value of protein

**Figure 6:**
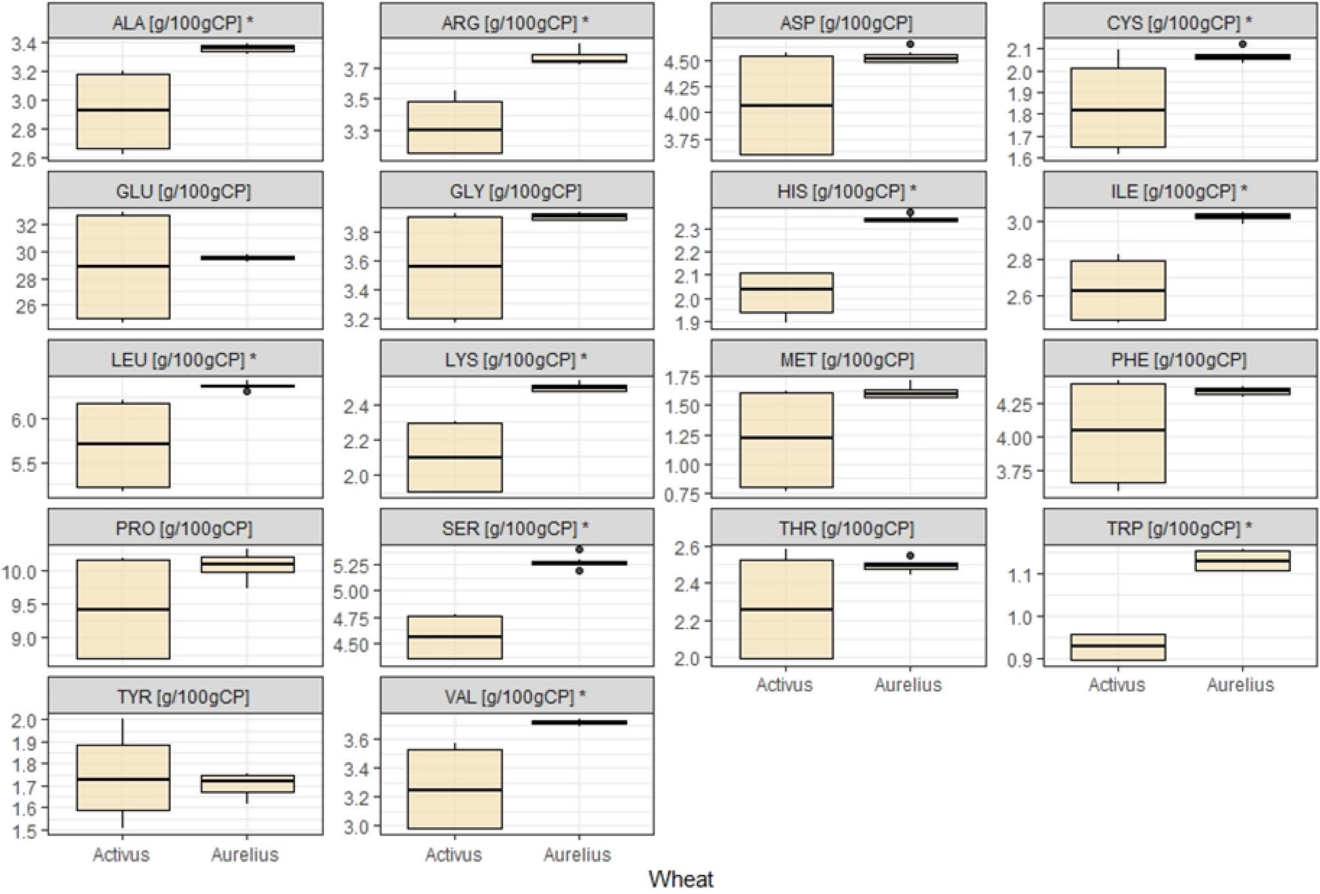
Amino acid content (g in 100 g of CP) of wheat grains protein, *statistically significant difference between cultivars of wheat.

The first AA limiting the nutritional value of the protein was LYS (Table 2S, Fig. 6). The average content of LYS in the tested grains was 2.10 (in cv. Activus)—2.50 (in cv. Aurelius) in g (100 g CP)^−1^. Thus, cv. Acticus was characterized by a significantly lower value of ca. 16% compared to cv. Aurelius. Therefore, the CS index for Activus was approx. 27%, while this value for the Aurelius cultivar was on average ca. 32% according to the adopted whole egg protein standards (Table 1, Fig. 6).

**Table 1:** Biological value of wheat grains protein (average values ± standard deviation).

Item	Wheat		
	Activus		Aurelius
Biological value of protein, %			
CS_WE_Lys	27.28 ± 2.71	≠	32.42 ± 0.33
∑ EAAI	54 ± 2.61	≠	62 ± 0.00
PER_1_	1.47 ± 0.20	≠	1.75 ± 0.01
PER_2_	1.93 ± 0.22	≠	2.25 ± 0.02
PER_3_	1.93 ± 0.09	≠	2.74 ± 0.04
BV	47 ± 2.85	≠	56 ± 0.00

≠ statistical differences.

CS (Chemical Score)—the limiting amino acid index; WE—the Whole Egg Protein Standards; EAAI—the Essential Amino Acids Index; the predicted PER (Protein Efficiency Ratio); BV—the Biological Value of the Protein.

The content of EAA was reflected in the calculated index of essential amino acids (EAAI), which was determined according to the adopted standard to be 54% for the Activus cultivar and 62% for Aurelius (*p* < 0.01). To approximate the nutritional value of wheat protein, the predicted BV and PER indexes were also calculated, and these produced statistically significantly higher values for Aurelius (Table 1).

Basic AA constituted 7.44–8.60 g (100 g CP)^−1^ with significant differences between cultivars (*p* < 0.001). Acidic AA accounted for 32.89–34.02 g (100 g CP)^−1^. Aromatic AA constituted 16.12–17.25 g (100 g CP)^−1^ (*p* < 0.05). The share of non-polar amino acids accounted for 33.65–37.54 g (100 g CP)^−1^ (*p* < 0.001). Polar AA accounted for 79.54–83.64 g (100 g CP)^−1^. The highest values for this parameters were observed in cv. Aurelius and the lowest in cv. Activus (Table 2S, Fig. 6).

The highest proportion of sulfur AA was observed in cv. Aurelius and the lowest in cv. Activus (Table 2S, Fig. 6). Therefore, the Aurelius cultivar was a better source of sulfur AA. The content of these AAs was over 3.6 g (100 g CP)^−1^. Moreover, cv. Aurelius proved to be significantly (α = 0.05) richer in TRP and HIS, LEU, ILE and VAL. Generally, wheat protein is found to be deficient in THR and ILE. In the tested samples, the Aurelius cultivar was characterized by higher values for these AAs compared to Activus.

**Figure 7:**
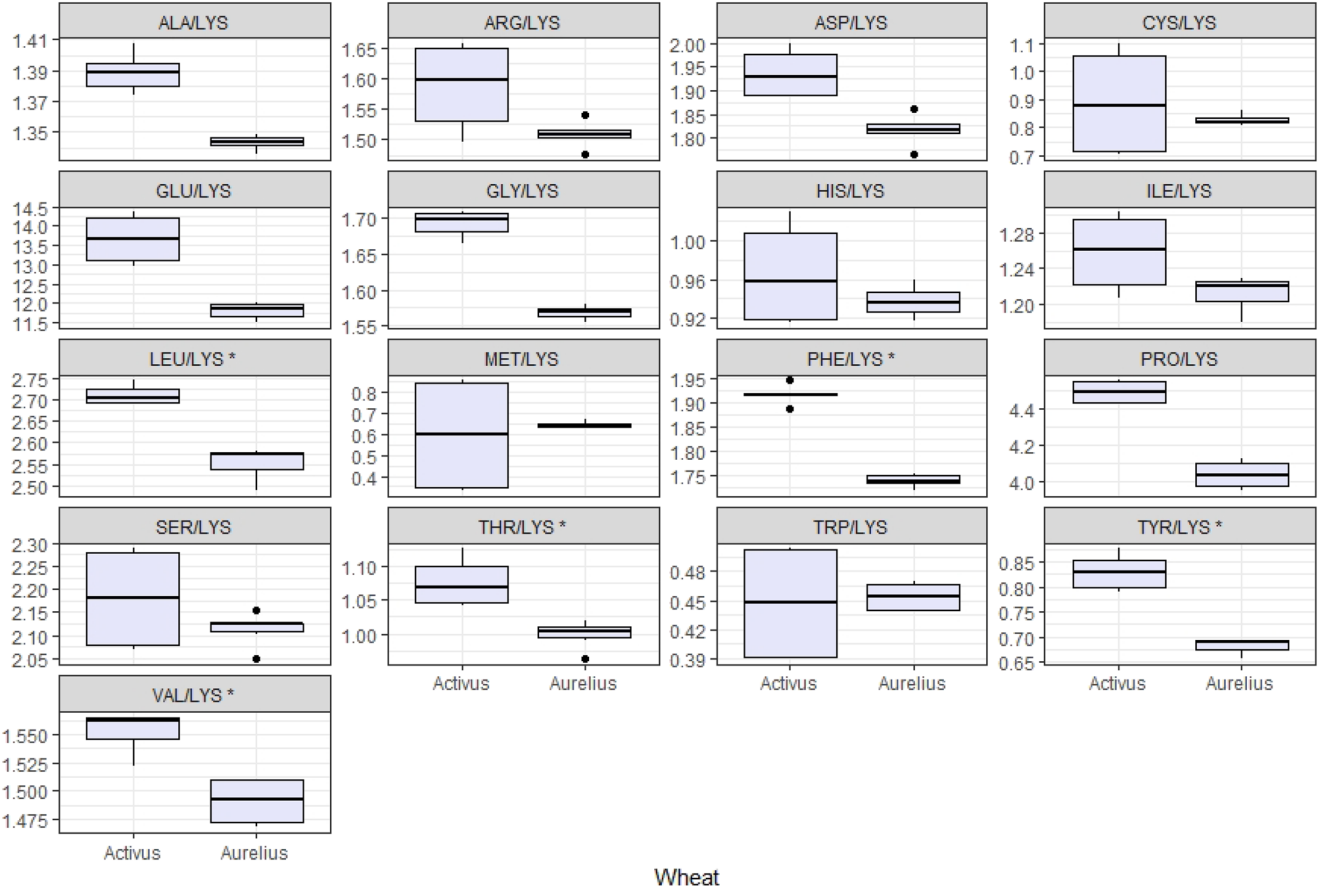
Essential amino acid profile relative to lysine in selected cultivars of winter wheat, *statistically significant difference between cultivars of wheat. *ASP* Aspartic acid, *THR* Threonine, *SER* Serine, *GLU* Glutamic acid, *PRO* Proline, *GLY* Glycine, *ALA* Alanine, *CYS* Cystine, *VAL* Valine, *MET* Methionine, *ILE* Isoleucine, *LEU* Leucine, *TYR* Tyrosine, *PHE* Phenyloalanine, *HIS* Histidine, *LYS* Lysine, *ARG* Arginine, TRP Tryptophan. The correct ratio of LYS:MET + CYS (MET):THR:TRP for weaners is 100:60:(30):62:18 (NŻŚ, 1993)^68^ and for growing rats it is 100:87(44):73:22 (NRC)^69^.

LYS is introduced in animal nutrition to properly balance diets. In practice, assuming a LYS value of 100%, the concentrations of other AAs were balanced accordingly. Relating the shares of AA to LYS, slightly higher shares of ILE/LYS (1.2–1.3) and THR/LYS (1.0–1.1) were found. There was also a higher share of VAL/LYS (1.5–1.6) and sulfur AA, i.e., (MET + CYS)/LYS (1.5), and next PHE/LYS (1.7–1.9). Noteworthy is LEU, the level of which was 2.5 times higher than LYS in the tested wheat grains (LEU/LYS = 2.6–2.7). The presented values were always higher for cv. Activus than for cv. Aurelius. The ARG to LYS ratio (ARG/LYS) varied from 1.51 to 1.59, and this was also higher for cv. Activus than for cv. Aurelius (*p* < 0.001) (Fig. 7).

### Macro- and microelement content of selected wheat cultivars

**Figure 8:**
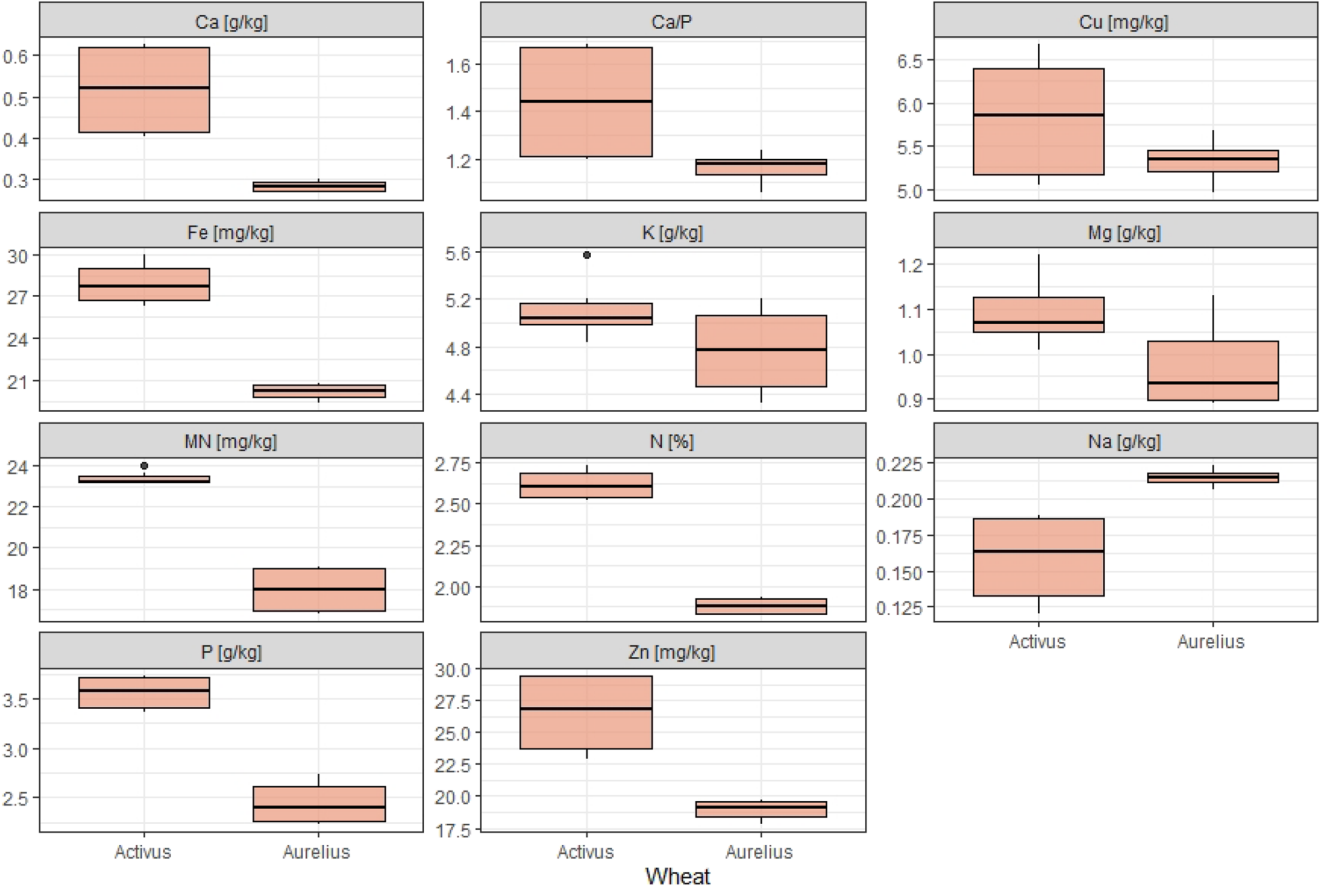
Macro- and microelements content in selected cultivars of winter wheat. *—statistically significant difference between cultivars of wheat.

Mineral compounds are important from the perspective of nutrients and technology. Winter wheat grain is a source of minerals, referred to as CA. Total nitrogen (N) content in wheat grain was 1.88% (cv. Aurelius)—2.61% cv. Activus (Table 3S, Fig. 8). However, the content of NPN was 0.08% in Aurelius and 0.13% in Activus, which accounted for 4% and 5% of TN, respectively. According to the available literature, the values for these parameters are mainly affected by nitrogen fertilization.

Significant differences were found between the tested cultivars in terms of the content of Ca, Na, and P. Significantly more Ca and P were found in the Activus cultivar, and significantly more Na in Aurelius (Table 3S, Fig. 8).

## Discussion

The results of the chemical composition and nutritional value analyses of the wheat cultivars varied and were dependent mainly on genetic properties. The moisture content in the wheat genotypes usually ranges from 7.8 to 14.8%^5,30^, which is in line with the results observed in our study, where the moisture content of wheat was on average 10.5%. The nutritional quality of grains is mainly determined by genetic traits of crops^31^. Environmental and soil conditions and technology^32^, mainly nitrogen fertilisation^33^, also affect grain traits. This thesis is also maintained in our study, where genetic characteristics determine nutritional value.

Wheat cultivars and conditions in a specific location can have an impact on the fiber content of wheat^7^. The content of CF affects nutritional value and impacts upon a cultivar’s use in animal nutrition. According to other authors^34^, wheat grain is characterized by a CF content of 10–14%. The grain of spring wheat contains significantly less CF than does cv. winter (from 10 to 22 g (kg DM)^−1^)^10^. The results obtained in this study indicate that CF value does not exceed 3%. However, the content of CF differed between the tested wheat cultivars (Aurelius—23 g kg^−1^, while in Activus—27 g kg^−1^). This result is consistent with the results of Rahmann and Kader^35^. Compared to other cereals, wheat contains less CF, which indicates its significant importance in the nutrition of monogastric animals.

In our studies concerning the content of CA, this value ranged from 14 (cv. Aurelius) to 16 g kg^−1^ (cv. Activus). These results were in agreement with research conducted by other authors^10,30^, where CA content in wheat ranged from 1.45 to 2.00%.

The tested wheat cultivars were characterized by a low fat (EE) content (11 g kg^−1^ in cv. Aurelius; 13 g kg^−1^ in cv. Activus). Biel and Maciorowski^10^ noted a higher fat content in the grain of Jara Korynta—23 g (kg DM)^−1^, while Markiza winter wheat had a value of 13 g (kg DM)^−1^. The share of fat in other studies also ranged between 10.6 and 25.8 g (kg DM)^−1^^36,37^. In addition to fat, carbohydrates present in cereal grains also represent a source of energy. Carbohydrates account for about 65–75% of the mature wheat grain^38^. The carbohydrate content of wheat in our research was 69.0–71.5%.

The CP content and AA balance determine the nutritional quality of wheat grain. The content of EAA in the protein is lower than the content of non-EAA, with GLU constituting the majority of wheat grain protein^39,40^. The grain protein content, which consists of gliadins and glutenins (storage proteins) and albumins and globulins (metabolic proteins), is an important determinant of wheat grain quality. It is intensively affected by genetic traits, cultivation practices, fertilizer application and irrigation^4^.

In the assessment of feed raw materials, attention is most often paid to the content of CP. The basic chemical compositions of the tested cultivars differed. The CP values were ca. 16% (cv. Activus) and ca. 12% (cv. Aurelius), and these were in accordance with the ranges given by other authors^10,36^. The research by these authors showed that the average CP content in wheat is approx. 13%^10^. However, other studies indicate a higher level of protein (176 g (kg DM)^−1^ spring cv. Corinth; 174 g (kg DM)^−1^ winter cv. Fregata)^41^. Wheat cultivars characterized by low protein values are referred to as feed and are used in feeding livestock. Different varieties of wheat are characterized by different CP values (from 12.95 to 18.14%) and AA compositions^42,43^. Rain abundance and low temperature from sowing to maturity result in lower protein content, whereas warm growing conditions result in high CP wheat^44^. The application of nitrogen fertilizer can have a significant effect on the composition of CP and AA in wheat grains. Increasing nitrogen application can significantly increase the CP content of wheat grains mainly by stimulating the accumulation of gliadins and glutenins^45,46^.

Analyzing the composition and sequence of AA, one should also pay attention to the amount of storage proteins (prolamine and glutelin), which determine the commercial quality of the grain. They are the essential components of the wheat gluten complex and determine the possibility of using the grain in human and animal nutrition. Glutamic acid and PRO are of significant structural importance in gluten proteins as together they constitute one half or more of the peptide-bound amino acids in them, whereas TRP content is the lowest of all AA profiled in gluten proteins^47^. The nutritional value of gluten proteins is quite low due to the low levels of essential amino acids such as LYS and TRP. Thus, more studies are needed to improve the nutritional quality of wheat by increasing its EAA content.

Amino acids (AAs) (especially EAAs, i.e., those which cannot be synthesized and therefore must be acquired through nutrition) determine the nutritional value of the CP^48^. The sum of all AA calculated in our study was similar (84 g (100 g BO)^−1^ for cv. Aurelius; 92 g (100 g BO)^−1^ for cv. Activus); unlike in the studies conducted by Biel and Maciorowski^10^, where the sum of all AA did not differentiate the cultivars and averaged 86 g (16 g N)^−1^. The non-EAA of cereals comprising ASP, SER, GLU, PRO, GLY, ALA and ARG, constituted 66% (Aurelius) and 67% (Activus) of the total AA. The level of EAA was lower than the level of non-EAA in the tested samples: 34% (Aurelius), 33% (Activus) of the total AA.

Glutamic acid was found to be the most abundant AA, with concentrations ranging from 28.8 g (cv. Activus) to 29.5 g (cv. Aurelius) (100 g of CP)^−1^. Thsee results are consistent with those of Jiang et al.^49^, who found GLU to be the most dominant AA with a concentration range of 24.79–37.05 g (100 g of CP)^−1^ in wheat. Other results collected in the available literature indicate a considerable difference in the amount of GLU in AA between the wheat cultivars: 30–37 g (100 g of CP)^−1^^4,50,51^. Glutamic acid is synthesized in the body and plays a major role in AA metabolism and in maintaining N balance in the body; this is essential in situations of stress and illness. Glutamic acid is also a well-established excitatory neurotransmitter in the central nervous system^30^.

In our study, the most limiting are LYS, TRP and MET of the EAA in wheat grains. In the studies by Konvalina et al.^52^, the LYS content in wheat grains averaged 3.85%, 3.37%, and 3.15% in six emmer wheat cultivars, four old bread wheat cultivars, and two bread wheat cultivars, respectively. Anjum et al.^50^ found that newly released wheat cultivars are nutritionally more superior than older ones, especially in terms of the percentages of EAA, particularly LYS.

The Aurelius cultivar is characterized by beneficial values in terms of having more AA per 100 g of CP (i.e., per 16 g N) than Activus (essential: LYS, CYS, TRP, HIS, LEU, ILE, VAL and endogenous: ARG, ALA, SER). Thus, the first AA limiting the biological value of wheat protein was LYS. The authors of other studies also indicate this AA as limiting^4,49,50^. The values obtained in our research, i.e., 2.10–2.5 g (100 g CP)^−1^ are consistent with the data reported by Biel and Maciorowski^10^. The second AA limiting the biological value of the protein was THR, followed by ILE and VAL. The values obtained by our study correspond with those from other studies^10,48,53^. Thus, the current amounts of LYS and THR in wheat are insufficient to cover the animals' needs for these AA. It is important to balance diets for animals with different sources of protein, which allows them to complement each other^54^.

It is generally accepted that wheat grain is a good source of sulfur AA. Bros^55^ reported that the level of sulfur AA in wheat was 3 g (16 g N)^−1^. In our own research, when assessing MET and CYS, we noted a lower level of these AA (especially CYS in cv. Aurelius). The level of MET in the total sulfur AA amounted to 44% in cv. Aurelius and 39% in cv. Activus. When using wheat in animal feed, low CYS levels can be partially compensated for by sufficient MET content (because MET is a precursor to CYS). Despite this, when balancing animal diets, it is necessary to ensure that there is no need to supplement the deficiency of MET and to improve its ratio to sulfur AA by adding raw materials rich in MET or its crystalline equivalent of AA. Similar relationships exist between PHE and TYR. In our own study, a lower level of TYR was noted in Aurelius. Because PHE is a precursor to TYR, low TYR levels may be partially compensated for by sufficient PHE content.

In addition, and based on our own research, by relating the mutual proportions of AA/LYS to the nutritional requirements of animals (pigs, rats), wheat protein is characterized by an excess of (MET + CYS)/LYS and THR/LYS. These values were higher for cv. Activus, and lower for cv. Aurelius. Therefore, cv. Activus could be a protein supplement for diets poor in these AAs. The relationship between TRP and LYS (TRP/LYS) also exceeded the recommendations given in standards for pigs (NŻŚ)^56^ and rats (NRC)^57^. In the tested samples, the ratio of TRP/LYS was 0.45, which is a desirable value in the nutrition of monogastric animals. Tryptophan regulates the metabolism of serotonin and dopamine. Therefore, it can influence the behavior of animals to some extent^58^. In the literature, it is reported that animals fed with a TRP-deficient diet consume less feed and grow more slowly^59^.

It is well known that LYS is primarily associated with growth, while ARG has a variety of effects on metabolism, including protein synthesis and immunity. There is an antagonistic relationship between ARG and LYS (they compete in intestinal absorption)^60,61^. An increase in the level of ARG in the diet entails an increase in the requirement for LYS^61^.Therefore, taking into account the low level of LYS in wheat and the relationship between ARG and LYS, special attention should be paid to the composition of diets and the ratio of raw materials to each other (especially in diets for broilers). More studies, however, are required to fully understand the role of ARG in this process.

The nutritional value of the protein was determined by EAAI (54–62%) and CS (30–36%). The calculated values of the indicators indicate Aurelius cultivar has better quality. The higher EAA as well as the total amino acid score may be attributed to the low amount of gluten proteins expressed in g kg^−1^. The protein quality of wheat grain was similar to the values obtained by Biel and Maciorowski^10^—CS_WE_ average 27.01% and EAAI_WE_ average 52.35%.

Predicted PER ratios in our study (1.75–2.74%) also confirm the superior nutritional value of Aurelius wheat protein. In the available literature, Biel and Maciorowski^10^ and Pèrez-Conesa et al.^62^ also estimated this parameter at the level of ca. 2%.

Suchowilska et al.^63^ demonstrated that the amounts of potassium and calcium did not vary significantly between cultivars. Grela^64^ claimed that potassium content was higher in spelt wheat compared to common wheat. In turn, Rachoń and Szumiło^65^ observed a higher content of these macroelements in grain of spelt and durum wheat compared to common wheat. In the study by Rachoń et al.^66^, the content of macroelements depended on the variety and also on cultivation technology. Spelt wheat was characterized by a higher content of phosphorus, calcium and magnesium, but a lower content of potassium. In our study, differences between cultivars were also visible in the case of N, Ca, Na and P with higher contents in Activus. High content differences for macro- and microelements in the grain depends not only on genotypes but also on location and year. In a study by Sabo et al.^67^, a higher content of N, P, K, Mg, Zn, Cu, and Mn, but not Fe, was found in grain of winter wheat genotypes cultivated in Donji Miholjac.

## Conclusions

The results from our study confirm that chemical composition depends on the genetic properties of cultivars. The Activus variety outperformed the Aurelius variety in many aspects. It had a higher total protein value, a higher proportion of true protein in crude protein, fat and energy, as well as a higher sum of essential amino acids. These results may indicate that the Activus cultivar could supplement compound feeds with these essential amino acids. However, despite the lower content of crude protein in the grain, Aurelius was observed to be more favorable in terms of the content of amino acid composition per 100 g of crude protein (especially essential amino acids). This favorable level of essential amino acids in Aurelius grains corresponded with the higher nutritional value of the protein (calculated on the basis of EAAI, PER and BV indexes).

Thus, better knowledge and understanding of the various physicochemical properties, protein quality and information on the amino acid composition will be beneficial not only to plant breeders but also to processors for the manufacturing of feed and food products.

### Supplementary Information

Supplementary Tables.

## Data Availability

All data generated or analyzed during this study are included in this published article. We declare that the plant material used for our study was purchased from Saatbau Polska Sp. z o.o. in Środa Śląska (Poland) as seed material. We did not use endangered plant species for the experiments. Experimental research and field studies on plants (either cultivated or wild), including the collection of plant material, complied with relevant institutional, national, and international guidelines and legislation.
