# Supplementary material for: Diversity of chemical composition and nutritional value in grain from selected winter wheat cultivars grown in south-western Poland
Source: Sci Rep. 2024 Feb 1;14:2630. doi: 10.1038/s41598-024-53094-0 (PMC10834549; doi:10.1038/s41598-024-53094-0)
Supplement: Supplementary file 1 — Supplementary Tables. [file 41598_2024_53094_MOESM1_ESM.docx]

**Table 1S.** Chemical composition and amino acid composition of wheat grains (average values ± standard deviation)

| Item | Wheat | | |
| --- | --- | --- | --- |
|  | Activus |  | Aurelius |
| **Nutrients, % · kg^-1^**: | | | |
| Dry matter  Crude ash  Crude protein (N x 6.25)  Crude fibre  Crude fat  Nitrogen free extracts  Gross energy, MJ  Gross energy, kcal | 90.86±2.02  1.61±0.06  16.34±0.56  2.67±0.01  1.30±0.13  68.93±1.62  18.63±0.01  4451.38±2.73 | ≠  ≠  ≠  ≠  ≠  ≠  ≠ | 88.08±0.05  1.43±0.05  11.75±0.32  2.27±0.42  1.09±0.13  71.54±0.83  18.31±0.06  4375.03±15.57 |
| **Amino acid, g · kg^-1^**: | | | |
| Lysine  Cystine  Methionine  Tryptophan  Arginine  Histidine  Phenylalanine  Tyrosine  Leucine  Isoleucine  Valine  Alanine  Glycine  Proline  Threonine  Serine  Glutamic acid | 3.44±0.45  2.99±0.25  1.95±0.65  1.51±0.01  5.44±0.48  3.30±0.26  6.60±0.87  2.85±0.40  9.33±1.16  4.31±0.42  5.34±0.66  4.78±0.61  5.83±0.82  15.44±1.80  3.72±0.59  7.46±0.61  47.27±8.28 |  | 2.93±0.05  2.43±0.08  1.89±0.04  1.33±0.06  4.43±0.08  2.75±0.08  5.10±0.10  2.00±0.03  7.49±0.21  3.55±0.11  4.37±0.13  3.94±0.07  4.60±0.10  11.83±0.28  2.93±0.08  6.20±0.16  34.65±1.13 |
| Ʃ EAA | 45.35±3.90 | ≠ | 36.78±0.90 |
| Ʃ AA | 138.25±17.54 | ≠ | 107.74±2.76 |
| **Participation of EAA in total AA, %** | 32.95±1.36 |  | 34.14±0.11 |

≠ statistical differences

*Ʃ AA - sum of all amino acids; Ʃ EAA - sum of essential aminoacids;*

**Table 2S.** Amino acid content (g in 100 g of CP) of wheat grains protein (average values ± standard deviation)

| Item | Wheat | | |
| --- | --- | --- | --- |
|  | Activus |  | Aurelius |
| **Amino acids, g · (100g of CP)^-1^** | | | |
| Essential amino acids:  Lysine  Cystine  Methionine  Tryptophan  Histidine  Phenylalanine  Tyrosine  Leucine  Isoleucine  Valine  Threonine | 2.10±0.21  1.83±0.21  1.20±0.44  0.93±0.03  2.02±0.10  4.03±0.40  1.74±0,19  5.69±0.53  2.64±0.18  3.26±0.30  2.27±0.29 | ≠  ≠  ≠  ≠  ≠  ≠  ≠ | 2.50±0.03  2.06±0.03  1.61±0.05  1.13±0.02  2.34±0.01  4.34±0.03  1.70±0.05  6.37±0.04  3.03±0.02  3.72±0.02  2.49±0.03 |
| Non-essential acids: | | | |
| Arginine  Alanine  Glycine  Proline  Serine  Glutamic acid | 3.32±0,19  2.92±0.28  3.56±0.39  9.43±0.80  4.56±0.23  28.81±4.15 | ≠  ≠  ≠ | 3.77±0.05  3.35±0.03  3.91±0.02  10.07±0.18  5.27±0.06  29.48±0.21 |
| **Crude protein, g** | 163.4±0.54 | ≠ | 117.5±0.31 |

≠ statistical differences

**Table 3S** Macro- and microelements content (average values ± standard deviation)

| Item | Wheat | | |
| --- | --- | --- | --- |
|  | Activus |  | Aurelius |
| **Macroelements, g · kg^-1^** | | | |
| N  Ca  Na  P  Mg  K | 2.61±0.09  0.52±0.12  0.16±0.03  3.56±0.17  1.09±0.08  5.11±0.25 | ≠  ≠  ≠  ≠ | 1.88±0.05  0.28±0.01  0.21±0.01  2.45±0.22  0.97±0.10  4.76±0.37 |
| **Microelements, mg · kg^-1^** | | | |
| Cu  Mn  Zn  Fe | 5.82±0.72  23.39±0.33  26.52±3.20  27.89±1.50 | ≠  ≠  ≠ | 5.33±0.25  17.95±1.15  18.92±0.80  20.21±0.59 |
| **Calcium to phosphorus ratio:** | 1.44±0.26 | ≠ | 1.16±0.07 |

≠ statistical differences
